# Characterization of two new degradation products of atorvastatin calcium formed upon treatment with strong acids

**DOI:** 10.3762/bjoc.15.206

**Published:** 2019-09-02

**Authors:** Jürgen Krauß, Monika Klimt, Markus Luber, Peter Mayer, Franz Bracher

**Affiliations:** 1 Department of Pharmacy – Center for Drug Research, Ludwig-Maximilians University Munich, Butenandtstr. 5-13, 81377 Munich, Germany; 2 Department of Chemistry, Ludwig-Maximilians University Munich, Butenandtstr. 5-13, 81377 Munich, Germany

**Keywords:** atorvastatin, crystal structure, cyclization, degradation products, fragmentation, stress test

## Abstract

Atorvastatin calcium (Lipitor^®^, Sortis^®^) is a well-established cholesterol synthesis enzyme (CSE) inhibitor commonly used in the therapy of hypercholesterolemia. This drug is known to be sensitive to acid treatment, but only little data has been published on the structures of the degradation products. Here we report the identification of two novel degradation products of atorvastatin, which are formed only under drastic acidic conditions. While treatment with conc. sulfuric acid led to a loss of the carboxanilide residue (accompanied by an expectable lactonization/dehydration process in the side chain), treatment with conc. aqueous hydrochloric acid gave a complex, bridged molecule under C–C-bond formation of the lactone moiety with the pyrrole, migration of the isopropyl group and loss of the carboxanilide residue. The novel degradation products were characterized by NMR spectroscopy, HRMS data and X-ray crystal structure analysis.

## Introduction

Over the past decades, the general trend toward globalization of the supply chains for active pharmaceutical ingredients has created new challenges for the authorities in ensuring the safety and quality of the drug supply [1]. Unprecedented impurities can appear, most likely if limited information is available about details (or alterations) of production processes of drugs. On the one hand, it is impossible to check drug substances routinely for all imaginable impurities, on the other hand it is desirable to identify as much as possible degradation products of drugs resulting from inappropriate exposition to potentially harmful conditions during production, manufacturing and storage. For being able to provide relevant data in a manageable time frame, two kinds of stress tests have found broad application: accelerated storage conditions (higher temperatures, higher humidity, …) typically provide reliable data on the stability of a drug, but are still time-consuming; on the other hand in “forced degradation experiments” the drug is submitted to more drastic conditions (e.g., strong acid or base, strong oxidant, very high temperature), and potential degradation products can be identified in very short time [2–3]. However, forced degradation experiments are highly artificial in nature, and thus one has to keep in mind that these extremely drastic conditions are prone to lead to results that might be out of proportion for daily quality control [3]. Nevertheless, knowledge about the outcome of stress tests under extreme conditions helps to get insight into the overall reactivity of drug substances.

Atorvastatin calcium (**1,** marketed as the trihydrate in Lipitor^®^, Sortis^®^), is a well-established drug for treatment of hypercholesterolemia [4]. This drug is monographed in the leading pharmacopoeias (Ph. Eur., USP), and a couple of impurities are listed there. Most of these impurities result from the synthesis process (stereoisomers, products resulting from impure starting materials or side reactions), and only one of these impurities, lactone **2**, is most likely a degradation product, resulting from acid-mediated lactonization of the 3,5-dihydroxyheptanoate side chain.

**Figure 1:**
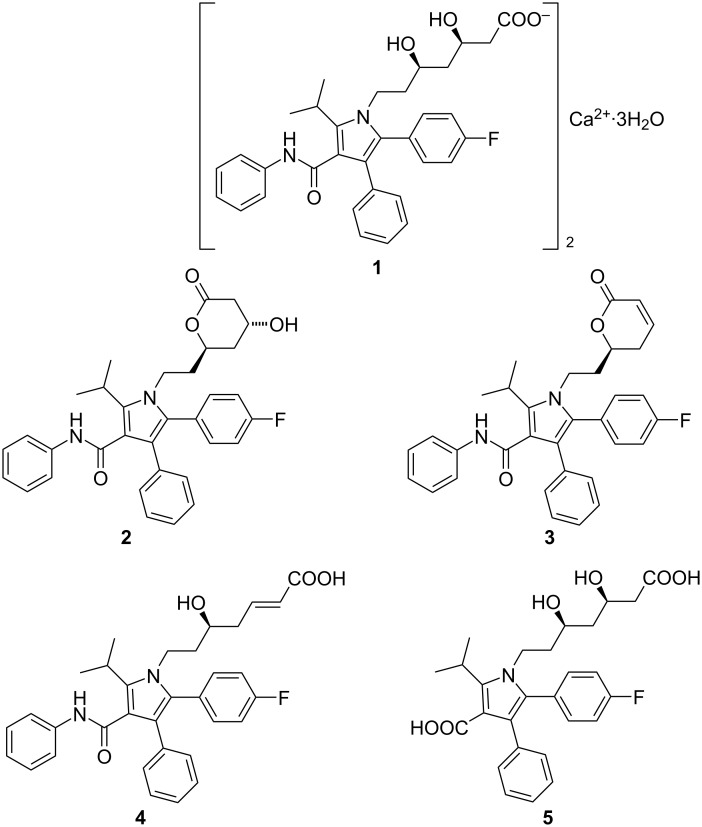
Atorvastatin calcium trihydrate (**1**) and previously published decomposition products arising from treatment with acids: lactone **2**, dehydrated lactone **3**, α,β-unsaturated carboxylic acid **4**, and carboxylic acid **5** (resulting from postulated anilide hydrolysis).

A couple of previous publications deal with stress tests on atorvastatin (and its salts), and an overview has been published by Sirén [5]. Hereby, atorvastatin was found to be sensitive to acidic, oxidative, photochemical and thermal stress. Acidic degradation of atorvastatin was reported to follow first order kinetics, but decomposition products were not characterized in this [6] and several other reports, which only determined the downsizing of the atorvastatin peak in HPLC after treatment with acid [7–10]. The most prominent decomposition product upon acidic treatment, compound **2**, results from lactonization of the 3,5-dihydroxyheptanoate side chain under moderately acidic conditions (0.1 M HCl) [11–14]. Shah et al. [15] identified six additional decomposition products upon treatment with 0.1 M HCl at 80 °C for 24 h, among which the dehydrated lactone **3** was dominating, accompanied by minor amounts of products arising from dehydration of the δ-hydroxy group and some epimers resulting from acid-catalyzed isomerization reactions. In contrast, Vukkum et al. [13] describe, besides lactones **2** and **3**, an α,β-unsaturated carboxylic acid **4**. Treatment under more drastic conditions (6 M HCl, reflux, 3 h) was reported to result mainly in hydrolysis of the anilide moiety to give carboxylic acid **5** [16–17] (Figure 1).

Here we report on the results of our investigations on the decomposition of atorvastatin calcium (**1**) under strongly acid conditions.

## Results and Discussion

### Stress tests

Since the lability of atorvastatin towards moderately acidic conditions is well-documented, we aimed at investigating the outcome of incubation with acids under more drastic conditions. Treatment of atorvastatin calcium trihydrate (**1**) with 2 M aqueous hydrochloric acid at room temperature (Table 1, entry 1) gave, in accordance with previous reports, only hydroxylactone **2** (55% yield). This outcome was confirmed by comparison with published NMR data [18–19]. At elevated temperature (reflux, 4 h; Table 1, entry 2) a mixture of lactone **2** and known unsaturated lactone **3** [15] (arising from acid-catalyzed dehydration of **2**) was obtained. Under even more drastic acidic conditions (refluxing with 6 M hydrochloric acid for 3 h, with 20% aqueous H_2_SO_4_ for 2 h, or with *p*-toluenesulfonic acid in toluene for 5 h; Table 1, entries 3–5) unsaturated lactone **3** was formed exclusively and in high to almost quantitative yields (Table 1).

**Table 1:** Acidic stress conditions and decomposition products formed.

Entry no.	Acidic conditions	Decomposition products (yield)			
		**2**	**3**	**6**	**7**

1	2 M HCl, 20 °C, 2 h	55%	–	–	–
2	2 M HCl, reflux, 4 h	65%	14%	–	–
3	6 M HCl, reflux, 3 h	–	70%	–	–
4	20% H_2_SO_4_, reflux, 2 h	–	>98%	–	–
5	*p*-toluenesulfonic acid, toluene, reflux, 5 h	–	95%	–	–
6	37% HCl, reflux, 5 h	–	–	96%	–
7	conc. H_2_SO_4_, 60 °C, 2 h	–	–	–	18%

**Scheme 1:**
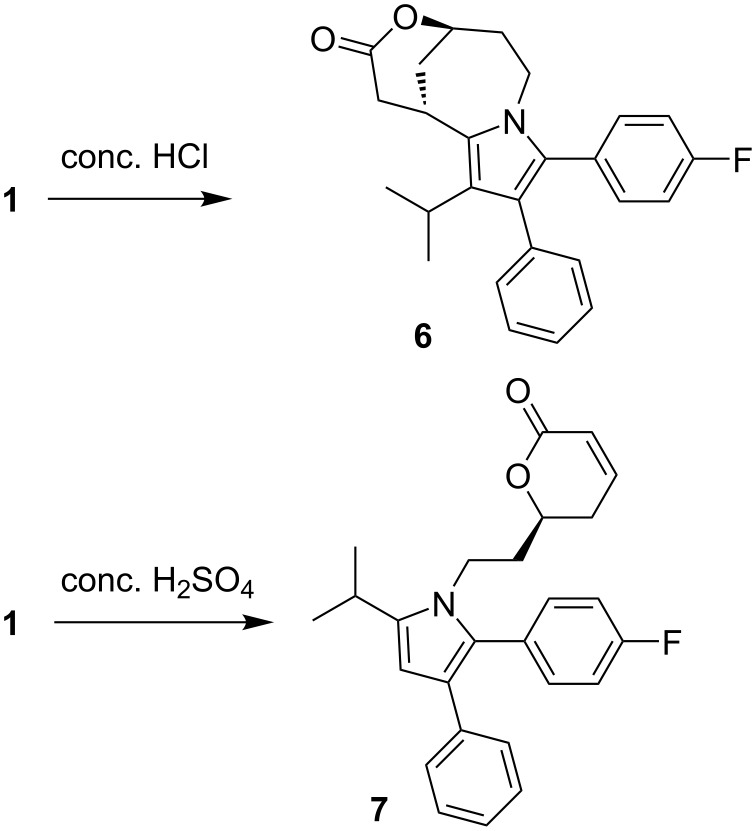
Formation of novel artefacts **6** and **7** under extremely strong acidic conditions.

**Figure 2:**
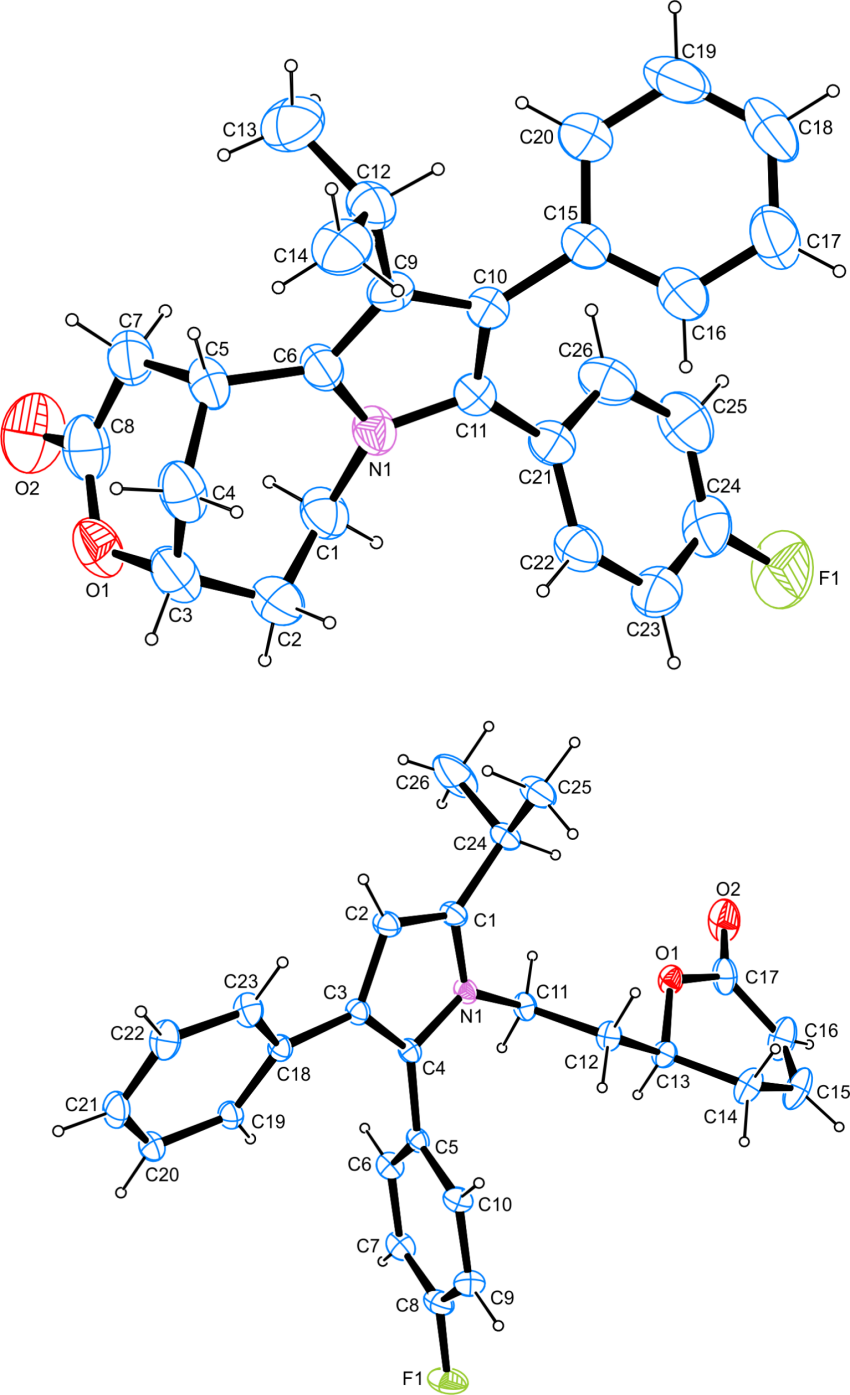
Top: Molecular structure of artefact **6**. Shown here is the molecular structure of one of three independent molecules in **6** drawn at the 50% ellipsoid probability level. Bottom: Molecular structure of artefact **7** (drawn at the 50% ellipsoid probability level).

When atorvastatin calcium trihydrate (**1**) was submitted to extremely strong acidic conditions by refluxing with concentrated (37%) aqueous hydrochloric acid (entry 6), a new product **6** was formed in almost quantitative yield. The ^1^H NMR analysis clearly indicated that the entire carboxanilide partial structure got lost under these conditions. However, no signal was observed which could be attributed to a C–H group at the pyrrole ring. The ^13^C NMR data showed one carbonyl resonance at 170.3 ppm, assignable to a lactone moiety. The HMBC experiment showed a cross peak between the proposed lactone carbonyl carbon and a neighboring CH-O group, confirming the lactone moiety, and the DEPT spectrum showed a new aliphatic methine resonance at 25.2 ppm. By HRESIMS mass data (found: 404.2020 for [M + H]^+^) a molecular formula of C_26_H_26_FNO_2_ was confirmed, excluding incorporation of HCl into this artefact. Finally, X-ray crystallography structure analysis (see Figure 2 and Supporting Information File 1) disclosed the structure of **6**, bearing a novel, bridged tricyclic 1,5-methanopyrrolo[1,2-*e*][1,5]oxazonin-3-one ring system (Scheme 1).

In contrast, submission of atorvastatin calcium trihydrate (**1**) to concentrated sulfuric acid for two hours at 60 °C (Table 1, entry 7) led to the degradation product **7** in low yield (18%) (Scheme 1). No further decomposition products could be isolated. Here, lactonization and dehydration steps in the side chain took place as observed before in the other acid treatments, however, under these extremely strong, virtually anhydrous acid conditions, the entire carboxanilide residue was removed to give the (*S*)-configured 4-unsubstituted pyrrole **7**, as exemplified by a typical CH resonance at 6.20 ppm in the ^1^H NMR spectrum. This structure was further confirmed by X-ray data (see Figure 2 and Supporting Information File 1).

### HPLC method for the detection of the novel impurities

**Figure 3:**
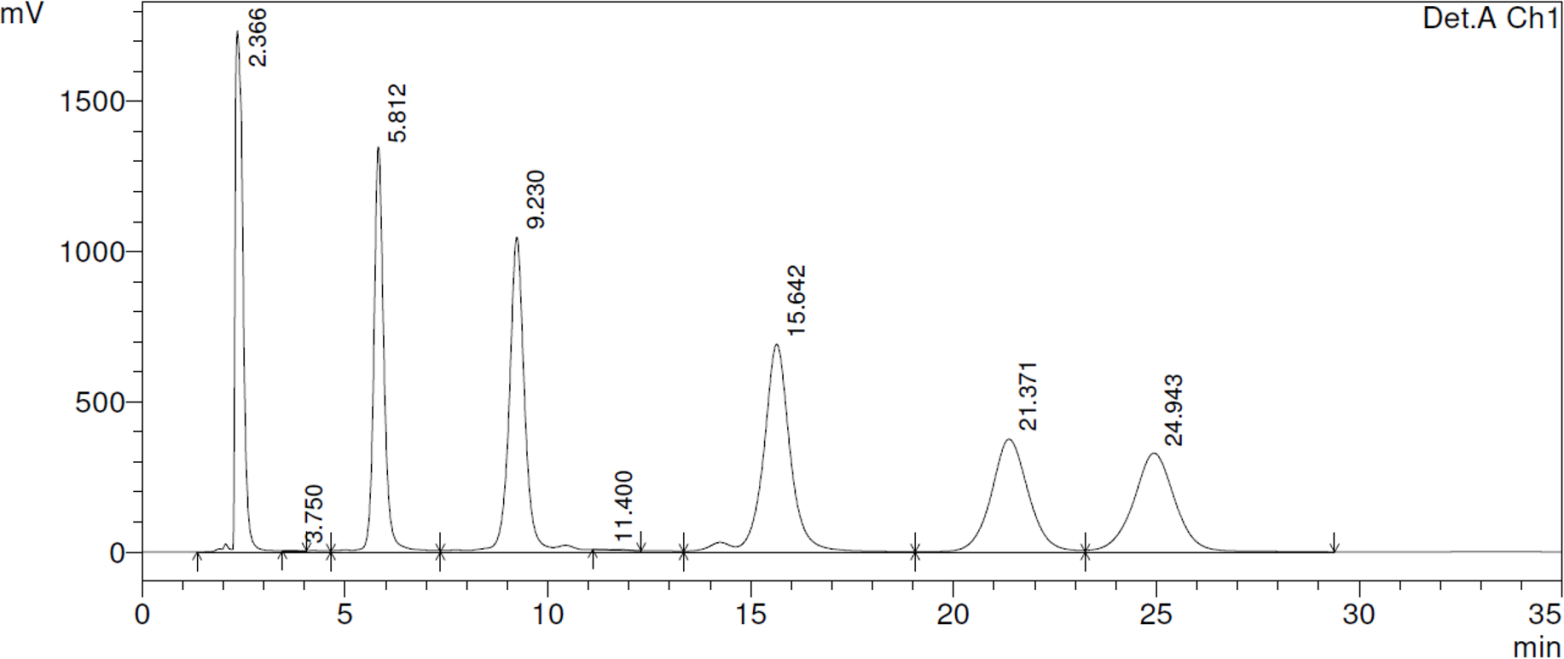
Separation of atorvastatin (**1**; retention time: 5.8 min) from the four decomposition products **2** (retention time: 9.2 min), **3** (retention time: 15.6 min), **6** (retention time: 21.4 min) and **7** (retention time: 24.9 min). Chromatogram obtained with a solution containing 10 mg each in 5.0 mL DMF (retention time 2.3 min), diluted 1:5 with the eluent buffer before injection.

In order to provide a convenient method for including our new findings into quality control of atorvastatin batches, we worked out an isocratic HPLC protocol, which prettily separates the four artefacts **2**, **3**, **6** and **7** from atorvastatin (**1**). This method uses an RP18 stationary phase (Eurospher 100–C18), isocratic elution with 0.01 M ammonium acetate buffer (pH 4)-acetonitrile 54:46 (v/v) at a flow rate of 1 mL/min at 40 °C, with UV detection at 246 nm (Figure 3).

## Discussion

In this investigation we first confirmed some pathways of decomposition of atorvastatin under acidic conditions. With dilute mineral acids at room temperature, atorvastatin is conveniently converted into the lactone **2** under retention at the C5–O bond of the aliphatic chain [13,20], whereas treatment under more drastic conditions (e.g., 6 M HCl or heating) causes expectable subsequent dehydration to give the unsaturated lactone **3** [13,15]. In contrast to previous reports [16–17] we could not find any indication for a cleavage of the carboxanilide partial structure to give free pyrrolecarboxylic acid **5** under treatment with 6 M HCl under reflux.

**Scheme 2:**
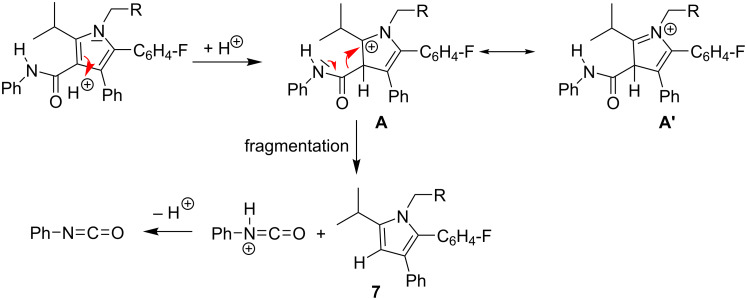
Proposed mechanism for the formation of desamidated product **7**.

However, upon treatment with concentrated sulfuric acid, lactonization/dehydration is accompanied by complete loss of the carboxanilide residue to give pyrrole **7**. Complete one-step removal of carboxamide residues from aromatic rings has been observed before in investigations of fragmentations of protonated species in mass spectrometry [21–22]. For benzanilides Tu [21] proposed a mechanism involving protonation of the amide oxygen, followed by 1,3 proton shift to the ring carbon next to the amide carbonyl group, followed by elimination of protonated phenyl isocyanate under re-aromatization. For the pyrrole derived substrate investigated here, even direct protonation at C-3 of the pyrrole by strong acid is most likely, due to the significant basicity of pyrroles. Delocalization of the positive charge (**A** ↔ **A’**) as shown in Scheme 2 will support the initial ring protonation step. The X-ray analysis of compound **7** revealed that the asymmetric center in the lactone ring is (*S*)-configured, indicating that once again the lactonization step took place with retention at the remaining stereocenter (the shift from (*R*) to (*S*) configuration is only a nomenclatory result of altered priorities of residues around the stereocenter upon dehydration). A comparable fragmentation has been observed in the CID (collision-induced dissociation) mass spectrum of atorvastatin, where the base peak observed at *m*/*z* 440 clearly corresponds to a loss of phenyl isocyanate [22].

**Scheme 3:**
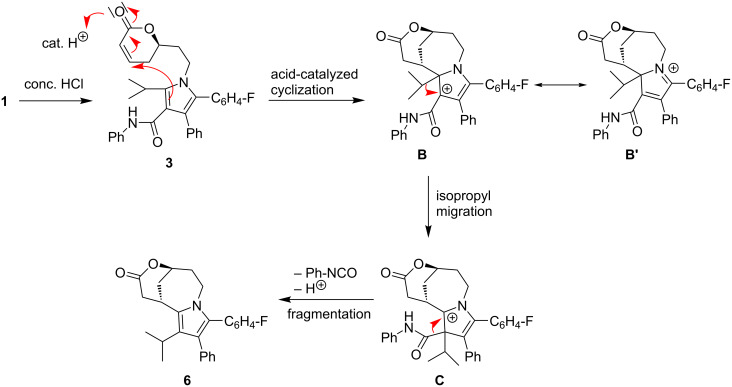
Proposed mechanism for the formation of bridged product **6** under cyclization, isopropyl migration and carboxanilide fragmentation.

In contrast, refluxing **2** with concentrated aqueous hydrochloric acid (37%) led to the formation of the complex, bridged product **6**. Most likely, this decomposition starts again with lactonization of the 3,5-dihydroxyheptanoate side chain, followed by dehydration to give the unsaturated lactone **3**. This hypothesis was confirmed by refluxing pure lactone **3** with 37% hydrochloric acid, resulting in clean conversion into **6** as well. In the following, this unsaturated lactone (Michael system) most likely performs an acid-mediated intramolecular attack at C-2 of the electron-rich pyrrole ring. In a cascade of subsequent reactions, the carboxanilide moiety at C-3 is eliminated and the isopropyl residue, originally located at C-2 of the pyrrole, is shifted to C-3, rendering the annulated, tetrasubstituted pyrrole **6**. The structure of **6** was confirmed by X-ray crystal structure analysis. However, most likely cleavage of the carboxanilide moiety (compare formation of **7** from atorvastatin with concentrated sulfuric acid) is not the initial step in the cascade of reactions leading from intermediate **3** to product **6**. In a control experiment, we treated compound **7** with 37% hydrochloric acid under the above mentioned conditions, but only a complex mixture of decomposition products was obtained, with no indication for formation of **6**. As the intermediate occurrence of a positive charge at C-2 of the pyrrole ring and a sp^3^-hybridized C-3 are most likely triggering the elimination of phenyl isocyanate from the pyrrole (see postulated mechanism shown in Scheme 2), we propose the formation of an intermediate **C**, which is formed via initial acid-mediated electrophilic attack of the unsaturated lactone at C-2 of the pyrrole ring. The resulting carbenium ion should be stabilized as shown in Scheme 3 (**B** ↔ **B’**). Subsequent shift of the isopropyl group from C-2 to C-3 then would give carbenium(-iminium) ion **C**, which can eliminate protonated phenyl isocyanate under formation of bridged pyrrole **6**. A comparable shift of the isopropyl group in atorvastatin has previously been observed only under oxidative conditions, giving rise to pyrrolidone-type degradation products [23].

## Conclusion

Atorvastatin calcium trihydrate (**1**) is known to be an acid-labile drug, and incubation with dilute mineral acids gives the known lactone **2**, which was further dehydrated to unsaturated lactone **3** under more drastic conditions. Treatment with very strong acids gave two hitherto unknown degradation products, whose structures were elucidated by NMR and crystal structure analysis. The bridged tricyclic product **6** was formed with concentrated hydrochloric acid, whereas lactone **7** resulted from treatment with concentrated sulfuric acid. We propose mechanisms for the formation of the novel artefacts **6** and **7** here. But it has to be mentioned that these new artefacts, which are formed only under extremely drastic conditions, are not likely to be relevant in terms of drug safety and control of impurities in launched atorvastatin batches.

## Supporting Information

File 1 Materials and methods; stress tests and analytical data of the products obtained thereby; details of characterization of **6** and **7** by X-ray data; crystallographic data for **6** and **7**, NMR spectra of artefacts **2**, **3**, **6**, and **7**.

File 2 checkCIF/PLATON report (Structure factors for artefacts **6** (wq033) and **7** (wv633)).
