# Supplementary material for: Characterization of two new degradation products of atorvastatin calcium formed upon treatment with strong acids
Source: Beilstein J Org Chem. 2019 Sep 2;15:2085–91. doi: 10.3762/bjoc.15.206 (PMC6753681; doi:10.3762/bjoc.15.206)
Supplement: File 1 — Materials and methods; stress tests and analytical data of the products obtained thereby; details of characterization of 6 and 7 by X-ray data; crystallographic data for 6 and 7, NMR spectra of artefacts 2, 3, 6, and 7. [file Beilstein_J_Org_Chem-15-2085-s001.pdf]

BEILSTEIN JOURNAL OF ORGANIC CHEMISTRY

Supporting Information

for

Characterization of two new degradation products of atorvastatin calcium formed upon treatment with strong acids

Jürgen Krauß, Monika Klimt, Markus Luber, Peter Mayer and Franz Bracher

Beilstein J. Org. Chem. **2019,** *15,* 2085–2091. doi:10.3762/bjoc.15.206

Materials and methods; stress tests and analytical data of the products obtained thereby; details of characterization of 6 and 7 by X-ray data; crystallographic data for 6 and 7, NMR spectra of artefacts 2, 3, 6, and 7

License and Terms: This is a supporting information file under the terms of the Creative Commons Attribution License (http://creativecommons.org/licenses/by/4.0). Please note that the reuse, redistribution and reproduction in particular requires that the authors and source are credited.
The license is subject to the *Beilstein Journal of Organic Chemistry* terms and conditions: (https://www.beilstein-journals.org/bjoc)

Table of contents:

1. Materials and methods

2. Stress tests and analytical data of the products obtained thereby

3. Details of characterization of **6** and **7** by X-ray data

4. Crystallographic data for **6** and **7**

5. NMR spectra of artefacts **2**, **3**, **6**, and **7**

6. References

1. Materials and methods

Atorvastatin calcium trihydrate (Ph. Eur.) was a gift of Prof. Dr. W. Frieß, LMU Munich. All solvents used were of HPLC grade or p.a. grade and/or purified according to standard procedures. Chemical reagents were purchased from Sigma-Aldrich (Schnelldorf, Germany) and Acros (Geel, Belgium). IR-spectra: Jasco FT/IR 4600 series (KBr pellet method); MS: Hewlett Packard MS-Engine, electron ionisation (EI) 70 eV, chemical ionisation (CI) with CH_4 (300 eV); MS spectra: Thermo Q Exactive GC Orbitrap or Finnigan MAT 95 spectrometer, HRESIMS spectra: Thermo Finnigan LTQ FT. NMR: Avance III HD 400 MHz Bruker BioSpin (^{1}H: 400 MHz, ^{13}C: 100 MHz); 500 MHz Avance III HD 500 MHz Bruker BioSpin (^{1}H: 500 MHz, ^{13}C: 125 MHz); melting points: Büchi Melting Point B-540 (not corrected); flash column chromatography (FCC): silica gel 60 (230–400 mesh, E. Merck, Darmstadt); HPLC: Shimadzu LC 10 pump, Shimadzu column oven CTO-10AS, Shimadzu autosampler SIL 10A, UV-detector Shimadzu LC 10 AS, column: Eurospher 100 – C18, 4 mm ID, (Knauer). Polarimeter: Perkin Elmer 241.

2. Stress tests and analytical data of the products obtained thereby

5-(4-Fluorophenyl)-1-(2-((2*R*,4*R*)-4-hydroxy-6-oxotetrahydro-2*H*-pyran-2-yl)ethyl)-2-isopropyl-*N*,4-diphenyl-1*H*-pyrrole-3-carboxamide (**2**)

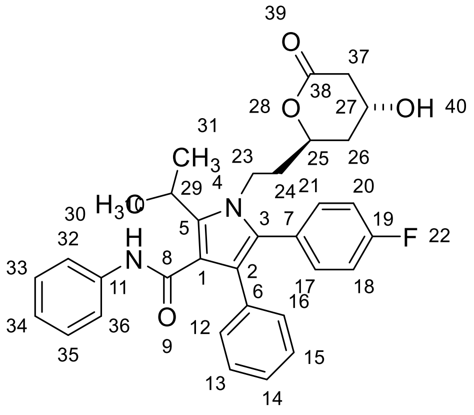

1.025 g (0.848 mmol) of atorvastatin calcium trihydrate (**1**) was dissolved in 50 mL 2 M aqueous hydrochloric acid and stirred for 2 h. Then the mixture was extracted with ethyl acetate (3 × 50 mL). The combined organic layers were dried over Na_2SO_4 and the solvent was evaporated. The residue was purified by flash column chromatography (ethyl acetate) to give 503 mg (55%) of **2** as a white solid. The same product could be isolated when **1** was treated with 2 M aqueous hydrochloric acid under reflux for 4 h to give 595 mg (65%) of **2**. M.p. 165 °C (ref. [1] 160 – 162

°C). $[\alpha]_D^{20}$ = + 19.6 (c 0.745, DMF); ^{1}H NMR (400 MHz, $CDCl_3$) δ 7.16 – 7.04 (m, 9 H, 9 arom. CH), 7.02 – 6.89 (m, 5 H, 5 arom. CH), 6.80 (s, 1 H, NH), 4.56-4.48 (m, 1 H, CH, 25-H), 4.34-4.27 (m 1 H, CH, 27-H), 4.22 (ddd, *J* = 14.9, 10.3, 5.0 Hz, 1 H, CH_2, 23-H), 4.03 (ddd, *J* = 14.9, 9.9, 5.7 Hz, 1 H, CH_2, 23-H), 3.62 – 3.45 (m, 1 H, CH, 29-H) , 2.66 (dd, *J* = 17.7, 4.8 Hz, 1 H, $CH_{2,}$ 37-H), 2.56 (ddd, *J* = 17.8, 3.5, 1.6 Hz, 1 H, $CH_{2,}$ 37-H), 1.86-1.74 (m, 1 H, CH_2, 24-H), 1.74 – 1.60 (m, 2 H, 2 CH_2, H-24, H-26), 1.56 – 1.49 (m, 1 H, CH_2, H-26), 1.58 – 1.49 (m, 6 H, 2 CH_3, 31-H, 30-H)). ^{13}C NMR (100 MHz, $CDCl_3$) δ 169.3 (CO, C-38), 164.9 (CO, C-8), 162.4 (d, *J* = 249.6 Hz, quat. C, C-19), 141.4 (quat. C), 138.2 (quat. C), 134.4 (quat. C), 133.1 (d, *J* = 9,0 Hz, 2 arom. CH), 130.4 (2 arom. CH), 128.7 (quat. C), 128.7 (2 arom. CH), 128.4 (2 arom. CH), 128.0 (d, *J* = 3.2 Hz, quat. C), 126.7 (arom. CH), 123.7 (arom. CH), 122.1 (quat. C), 119.7 (2 arom. CH), 115.7 (quat. C), 115.6 (d, *J* = 20.76 Hz, 2 arom. CH), 73.0 (CH, C-25), 62.5 (CH, C-27), 40.8 (CH_2, C-23), 38.5 (CH_2, C-37), 37.2 (CH_2, C-24), 35.7 (CH_2, C-26), 26.2 (CH, C-29), 22.0 (CH_3), 21.7 (CH_3). IR (KBr): ν (cm^{-1}) = 3405, 2963, 2930, 1724, 1653, 1507, 1437, 1314, 1225, 1156, 1110, 1074, 1031, 1011, 915, 885, 808, 754, 694, 617. HR-ESI-MS calcd. for $C_{33}H_{34}FN_2O_4^+$ $[M+H]^+$: 541.2497, found: 541.2498.

(*S*)-5-(4-Fluorophenyl)-2-isopropyl-1-(2-(6-oxo-3,6-dihydro-2*H*-pyran-2-yl)ethyl)-*N*,4-diphenyl-1*H*-pyrrole-3-carboxamide (3)

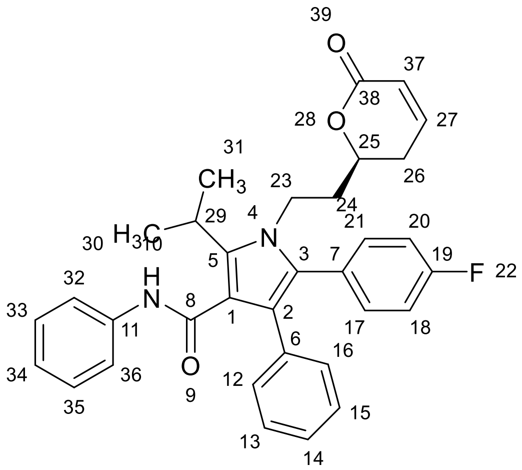

1.00 g (0.841 mmol) of atorvastatin calcium trihydrate (**1**) was dissolved in 200 mL toluene, 200 mg (1.07 mmol) *p*-toluenesulfonic acid were added and the suspension was refluxed for 5 h at a water separator. The solvent was evaporated, the residue dissolved in 50 mL aqueous 2 M hydrochloric acid and extracted with ethyl acetate (3 × 50 mL). The combined organic layers were dried over Na_2SO_4, the solvent was evaporated and the residue was purified by flash column chromatography (isohexane/ethyl acetate 1:1) to give 795 mg (95%) of **3** as an amorphous solid with an extremely broad melting interval (168–195 °C). $[\alpha]_D^{20}$ = −49.6 (c 0.74, DMF). The same

product could be isolated in >98% yield after refluxing atorvastatin calcium trihydrate (**1**) for two hours with 20% aqueous sulfuric acid. ^{1}H NMR (500 MHz, $CDCl_3$) δ 7.21 – 7.11 (m, 9 H, 9 arom. CH), 7.06 (d, *J* = 7.7 Hz, 2 H, 2 arom. CH), 7.04 – 6.94 (m, 3 H, 3 arom. CH), 6.87 (s, 1 H, NH), 6.78 (ddd, *J* = 8.9, 5.7, 2.6 Hz, 1 H, -CH=, 27-H), 6.00 – 5.94 (m, 1 H, CH=, 37-H), 4.29-4.18 (m, 2 H, CH, CH_2, 23-H, 25-H), 4.10-4.00 (m, 1 H, CH_2, 23-H), 3.63-3.51 (m, 1 H, CH, 29-H), 2.22 - 2.06 (m, 2 H, CH_2,26-H), 2.04 - 1.92 (m, 1 H, CH_2, H-24), 1.84 – 1.73 (m, 1 H, CH_2, H-24), 1.57-1.51 (m 6 H, 2 CH_3, 30-H, 31-H). ^{13}C NMR (125 MHz, $CDCl_3$) δ 164.7 (CO), 163.5 (CO), 162.3 (d, *J* = 247.8 Hz, quat. C), 144.7 (CH=, C-27), 141.3 (quat. C), 138.3 (quat. C), 136.35 (quat. C), 133.1 (d, *J* = 8.1 Hz, 2 arom. CH), 130.4 (2 arom. CH), 128.7 (2 arom. CH), 128.4 (2 arom. CH), 128.1 (d, *J* = 4.2 Hz, quat. C), 127.5 (quat. C), 126.7 (arom. CH), 123.6 (arom. CH), 122.1 (quat. C), 121.3 (CH=, C-37), 119.6 (2 arom. CH), 115.7 (d, *J* = 21.4 Hz, 2 arom CH), 75.2 (CH, C-25), 40.6 (CH_2, C-23), 36.4 (CH_2, C-24), 29.1 (CH_2, C-26), 26.2 (CH, C-29), 22.0 (CH_3), 21.7 (CH_3). IR (KBr): ν (cm^{-1}) = 3402, 2959, 2928, 1722, 1665, 1595, 1526, 1242, 1155, 1043, 816, 753, 693. MS (EI): m/z = 522 (M^+, 10), 430 (100). HR-MS calcd. for $C_{33}H_{31}O_3N_2F^{+\cdot}$ [$M^{+\cdot}$]: 522.2313, found: 522.2313.

(1*S*,5*R*)-9-(4-Fluorophenyl)-11-isopropyl-10-phenyl-1,2,6,7-tetrahydro-3*H*,5*H*-1,5-methanopyrrolo[1,2-*e*][1,5]oxazonin-3-one (6)

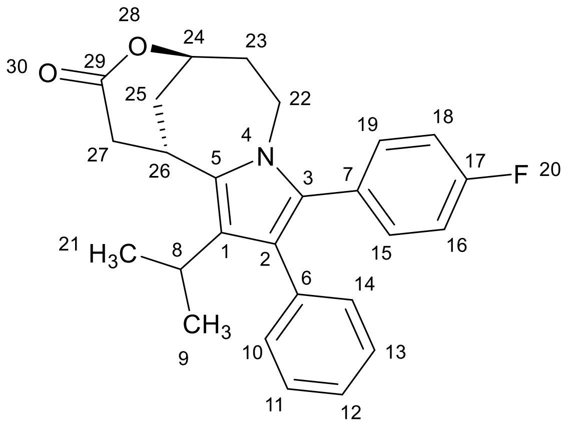

2.41 g (2.00 mmol) of atorvastatin calcium trihydrate (**1**) was dissolved in 50 mL concentrated (37%) aqueous hydrochloric acid and refluxed for 5 h. After addition of 40 mL water the mixture was extracted with ethyl acetate (3 × 40 mL). The combined organic layers were dried over Na_2SO_4 and the solvent was evaporated. The residue was purified by flash column chromatography (isohexane-ethyl acetate 1:1) to give 775 mg (96%) of **6** as a white solid. M.p.: 208 °C. $[\alpha]_D^{20}$ = -68.7 (c 2.07, DMF); ^{1}H NMR (500 MHz, $CDCl_3$) δ 7.16 – 7.03 (m, 3 H, 3 arom. CH), 7.02 – 6.91 (m, 4 H, 4 arom. CH), 6.90 – 6.79 (m, 2 H, 2 arom. CH), 5.09 – 5.03 (m, CH, 24-

H), 3.95 (ddd, *J* = 15.6, 4.8, 2.8 Hz, 1 H, CH_2 22-H), 3.87 (dd, *J* = 9.3, 5.8 Hz, 1 H, CH, 26-H), 3.63 (dd, *J* = 15.6, 12.2 Hz, 1 H, CH_2, 22-H), 3.06 (dd, *J* = 19.5, 10.2 Hz, 1 H, CH_2), 2.92 – 2.78 (m, 1 H, CH, 8-H), 2.51 – 2.41 (m, 1 H, CH_2, 27-H), 2.35 – 2.20 (m, 1 H, CH_2, 23-H), 2.06 – 1.96 (m, 1 H, CH_2. 27-H), 1.94 - 1.86 (m, 1 H, CH_2), 1.10 (m, , 6 H, 2 CH_3, 21-H, 9-H). ^{13}C NMR (100 MHz, $CDCl_3$) δ 170.3 (CO, C-29), 161.8 (d, *J* = 247 Hz, quat. C, C-17), 136.5 (quat. C), 132.7 (d, *J* = 7.9 Hz, 2 arom. CH), 131.7 (quat. C), 131.1 (2 arom. CH), 129.9 (quat. C), 128.3 (d, *J* = 3.4 Hz, quat. C), 127.6 (2 arom. CH), 125.7 (arom. CH), 125.4 (quat. C), 122.3 (quat. C) 115.1 (d, *J* = 21.24 Hz, 2 arom. CH), 77.3 (CH, C-24), 39.6 (CH_2, C-22), 34.7 (CH_2), 34.3 (CH_2), 30.6 (CH_2, C-27), 26.5 (CH), 25.2 (CH), 24.2 (CH_3), 24.0 (CH_3). IR (KBr): ν (cm^{-1}) = 2961, 2939, 1742, 1719, 1602, 1526, 1508, 1469, 1347, 1241, 1222, 1087, 1057, 846, 704. HR-ESI-MS calcd. for $C_{26}H_{27}FNO_2^+$ $[M+H]^+$: 404.2020, found: 404.2020.

(*S*)-6-(2-(2-(4-Fluorophenyl)-5-isopropyl-3-phenyl-1*H*-pyrrol-1-yl)ethyl)-5,6-dihydro-2*H*-pyran-2-one (7)

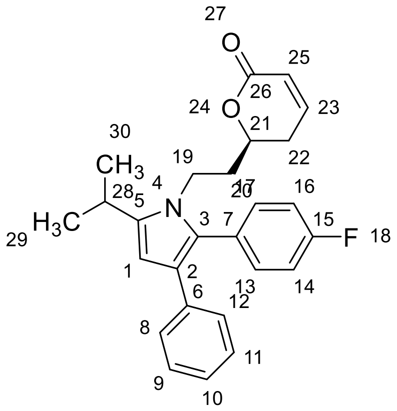

992 mg (0.821 mmol) of atorvastatin-calcium trihydrate (**1**) was dissolved in 20 mL concentrated sulfuric acid and the mixture was stirred for 2 h at 60 °C. The mixture was carefully diluted with 40 mL ice-water and extracted with ethyl acetate (3 × 40 mL). The combined organic layers were dried over Na_2SO_4, the solvent was evaporated and the residue purified by flash column chromatography (isohexane-ethyl acetate 1:1) to give 117 mg (18%) of **7** as a grey solid. $[\alpha]_D^{20}$ = -46.7 (c 0.51, DMF); ^1H NMR (400 MHz, $CDCl_3$) δ 7.30 – 7.24 (m, 2 H, 2 arom. CH), 7.19 – 6.99 (m, 7 H, 7 arom. CH), 6.77 (ddd, *J* = 9.7, 5.9, 2.6 Hz, 1 H, CH=, 23-H), 6.20 (s, 1 H, arom. CH, 1-H), 5.96 (ddd, *J* = 9.7, 2.6, 1.0 Hz, 1 H, CH=, 25-H), 4.21 – 4.06 (m, 2 H, CH, CH_2, 21-H, 19-H), 3.98 (ddd, *J* = 14.9, 9.1, 6.5 Hz, 1 H, CH_2, 19-H), 3.02 (m, 1 H, CH, 28-H), 2.17 – 1.93 (m, 2 H, CH_2), 1.86 (dtd, *J* = 13.9, 8.9, 5.0 Hz, 1 H, CH_2), 1.74 – 1.57 (m, 1 H, CH_2), 1.39 – 1.29 (m, 6 H, 2 CH_3, 29-H, 30-H). ^{13}C NMR (100 MHz, $CDCl_3$) δ 163.7 (CO, C-26), 162.3 (d, *J* = 248.4 Hz, Hz, quat. C, C-15), 144.7 (-CH=, C-23), 140.6 (quat. C), 136.3 (quat. C), 132.9 (d, *J* = 8.2 Hz, 2 arom.

CH), 129.6 (d, J = 3.3 Hz, quat. C), 128.0 (2 arom. CH), 127.7 (quat. C), 127.5 (2 arom. CH), 125.0 (arom. CH), 122.6 (quat. C), 121.3 (-CH=, C-25), 115.9 (d, J = 16.0 Hz, 2 arom. CH), 103.9 (arom. CH, C-1), 75.1 (CH, C-21), 39.5 (CH_2, C-19), 36.2 (CH_2, C-20), 29.1 (CH_2, C-22), 25.7 (CH, C-28), 23.8 (CH_3), 23.5 (CH_3). MS (EI) m/z = 403 (M^+, 100), 388 (59), 360 (19), 276 (56). IR (KBr): ν (cm^{-1}) = 2962, 2925, 1720, 1599, 1523, 1507, 1348, 1240, 1222, 1087, 851, 695. HR-MS calcd. for $C_{26}H_{26}FNO_2$ [M^+]: 403.1948, found: 403.1951.

3. Details of characterization of 6 and 7 by X-ray data

The data of **6** have been collected at room temperature on a Bruker D8 Quest IµS diffractometer while those of **7** have been collected at 112 K on a Bruker D8 Venture TXS diffractometer. Mo Kα radiation monochromated by multilayer mirror optics was applied in both experiments. The frames were integrated with the Bruker SAINT software package [2] using a narrow-frame algorithm. Data were corrected for absorption effects using the Multi-Scan method of SADABS [3]. The structures were solved and refined using the Bruker SHELXTL Software Package [4]. The hydrogen atoms were calculated in ideal geometry riding on their parent atoms. The data quality of **6** did not allow the determination of the correct absolute structure (the randomly chosen structure solution used to draw Figure 2 in the manuscript shows (*R*)-configuration at C3 and (*S*) configuration at C5 (numbering used in the graphic in Figure 2), as well as the stereocenters of the two independent molecules not displayed herein: C29, C31, C55 and C57). Hence, this structure has been refined as perfect inversion twin. However, the data of **7** led to a Flack parameter of 0.1(2) indicating the correct absolute structure as that with an (*S*)-configured stereocenter (data have been collected up to a resolution of 0.60 Å). The figures were drawn at the 50% ellipsoid probability level (ORTEP [5]).

Crystallographic data have been deposited with the Cambridge Crystallographic Data Centre (CCDC 1907390 (**6**), 1907391 (**7**)). These supplementary crystallographic data can be obtained free of charge from The Cambridge Crystallographic Data Centre via https://www.ccdc.cam.ac.uk/structures/.

4. Crystallographic data for 6 and 7

Table S1

	**6**	**7**
CCDC	1907390	1907391
net formula	$C_{26}H_{26}FNO_2$	$C_{26}H_{26}FNO_2$
M_r/g mol^{-1}	403.48	403.48
crystal size/mm	0.100 × 0.030 × 0.020	0.100 × 0.070 × 0.060
crystal system	orthorhombic	orthorhombic
space group	'P 21 21 21'	'P 21 21 21'
a/Å	9.6001(8)	9.7398(4)
b/Å	17.4122(16)	10.4787(5)
c/Å	38.547(4)	20.4221(9)
α/°	90	90
β/°	90	90
γ/°	90	90
V/Å^3	6443.5(10)	2084.29(16)
Z	12	4
calc. density/g cm^{-3}	1.248	1.286
μ/mm^{-1}	0.084	0.087
transmission factor range	0.96–1.00	0.96–0.99
refls. measured	77036	48775
R_{int}	0.1383	0.0545
mean σ(I)/I	0.0889	0.0418
θ range	2.339–25.350	3.484–36.313
observed refls.	7567	8541
x, y (weighting scheme)	0.0342, 2.1173	0.0756, 0.0638
Flack parameter	0.5	0.1(2)
refls in refinement	11793	10078
parameters	818	273
restraints	0	0
$R(F_{obs})$	0.0667	0.0524
$R_w(F^2)$	0.1283	0.1307
S	1.038	1.082
shift/error$_{max}$	0.001	0.001
max electron density/e Å^{-3}	0.172	0.490
min electron density/e Å^{-3}	−0.190	−0.287

5. NMR spectra

5-(4-Fluorophenyl)-1-(2-((2*R*,4*R*)-4-hydroxy-6-oxotetrahydro-2*H*-pyran-2-yl)ethyl)-2-isopropyl-*N*,4-diphenyl-1*H*-pyrrole-3-carboxamide (2)

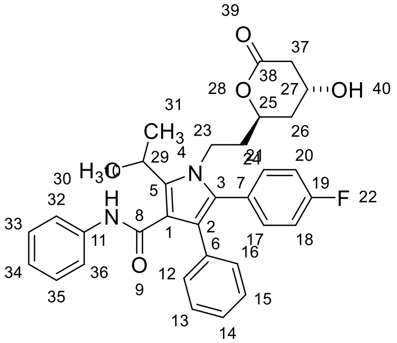

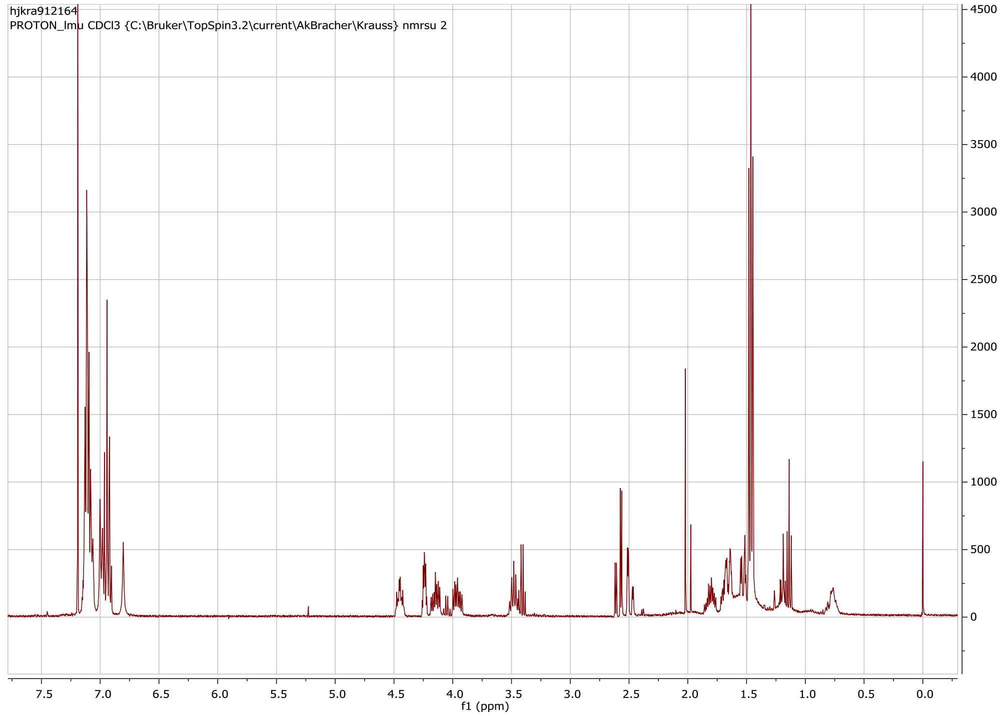

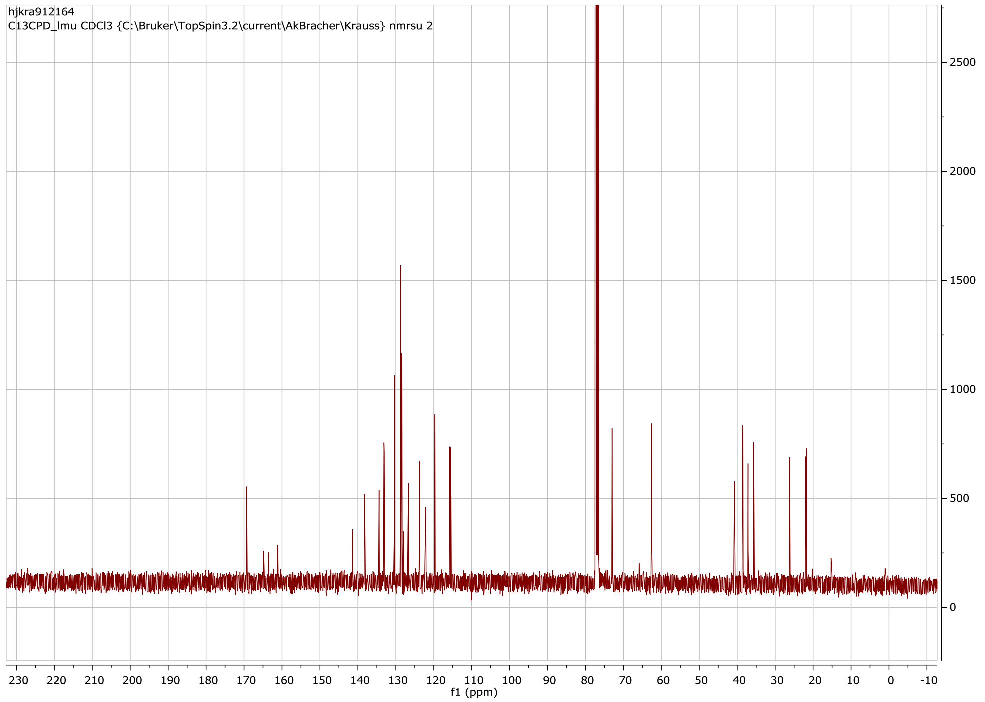

(*S*)-5-(4-Fluorophenyl)-2-isopropyl-1-(2-(6-oxo-3,6-dihydro-2*H*-pyran-2-yl)ethyl)-*N*,4-diphenyl-1*H*-pyrrole-3-carboxamide (3)

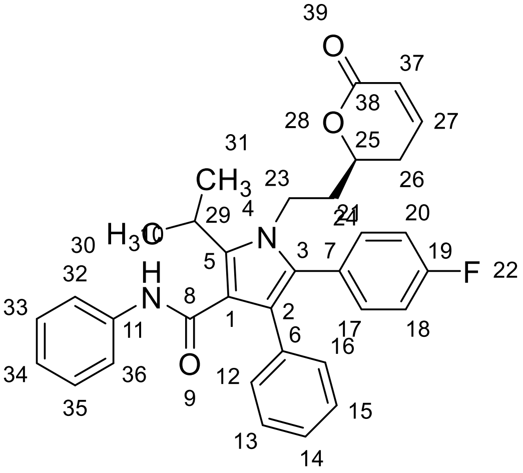

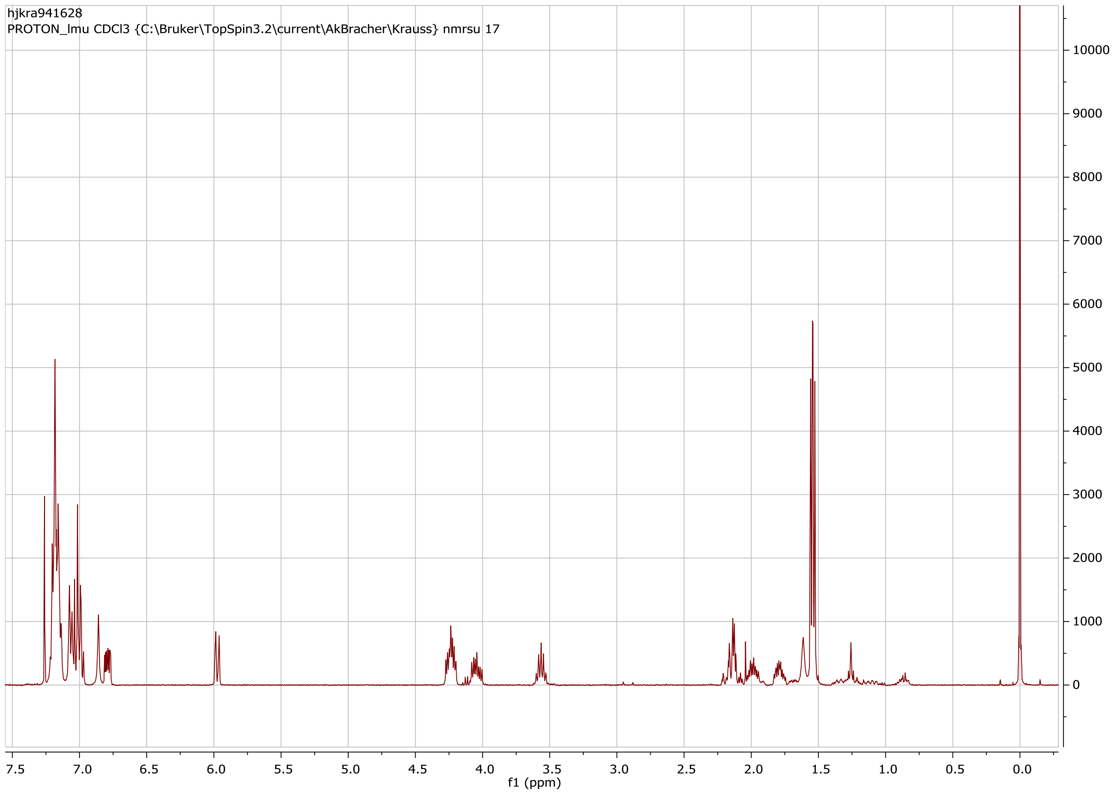
hjkra941628
PROTON_lmu CDCl3 {C:\Bruker\TopSpin3.2\current\AkBracher\Krauss} nmrsu 17
f1 (ppm)

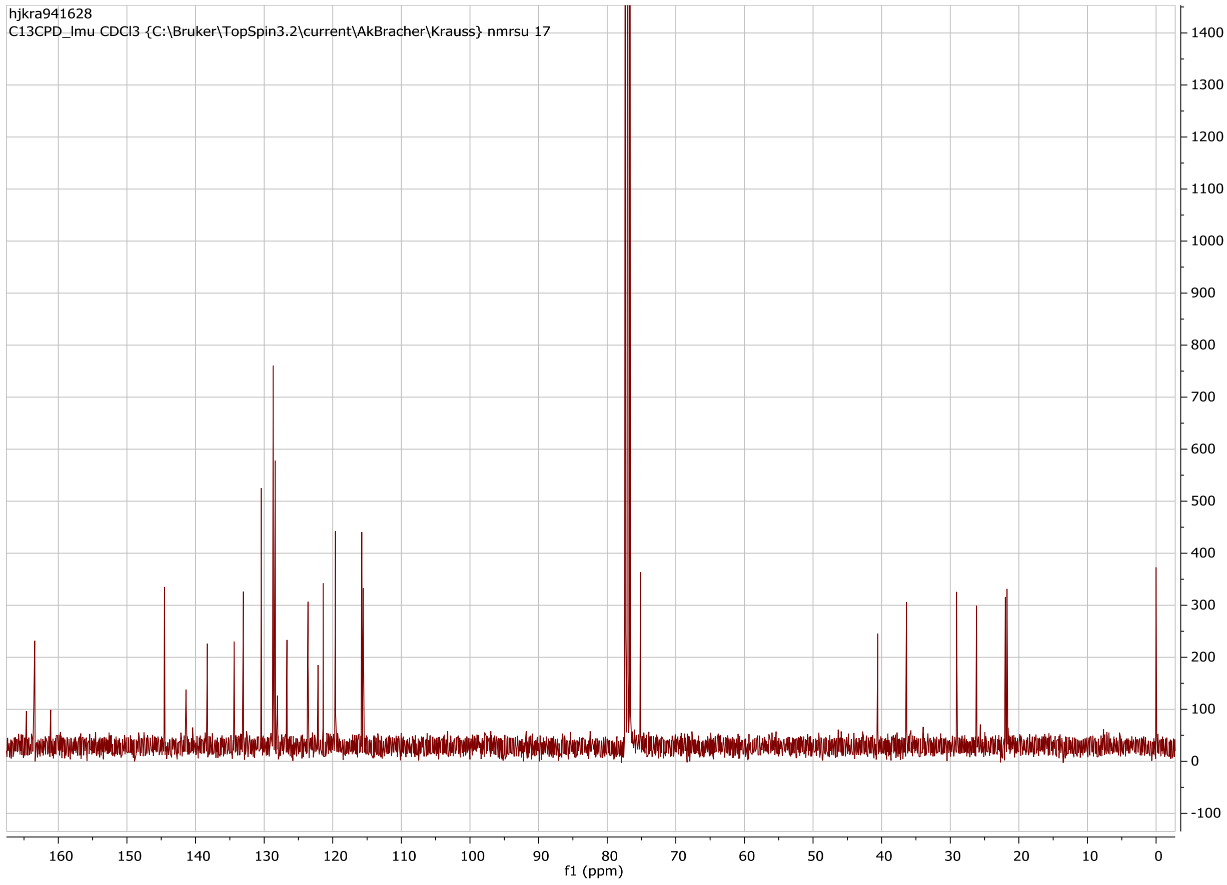
hjkra941628
C13CPD_lmu CDCl3 {C:\Bruker\TopSpin3.2\current\AkBracher\Krauss} nmrsu 17
f1 (ppm)

(1*S*,5*R*)-9-(4-Fluorophenyl)-11-isopropyl-10-phenyl-1,2,6,7-tetrahydro-3*H*,5*H*-1,5-methanopyrrolo[1,2-*e*][1,5]oxazonin-3-one (6)

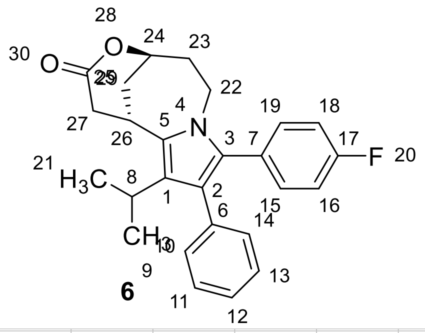

6

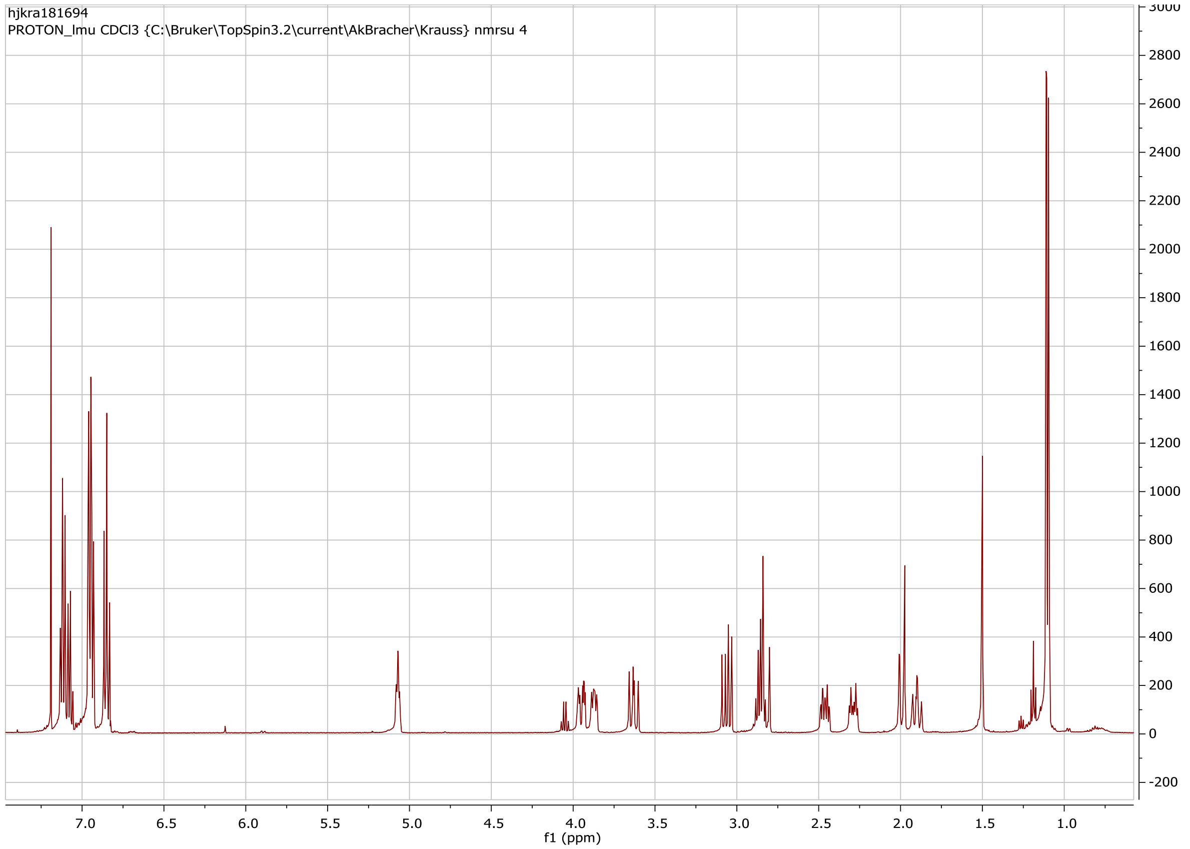

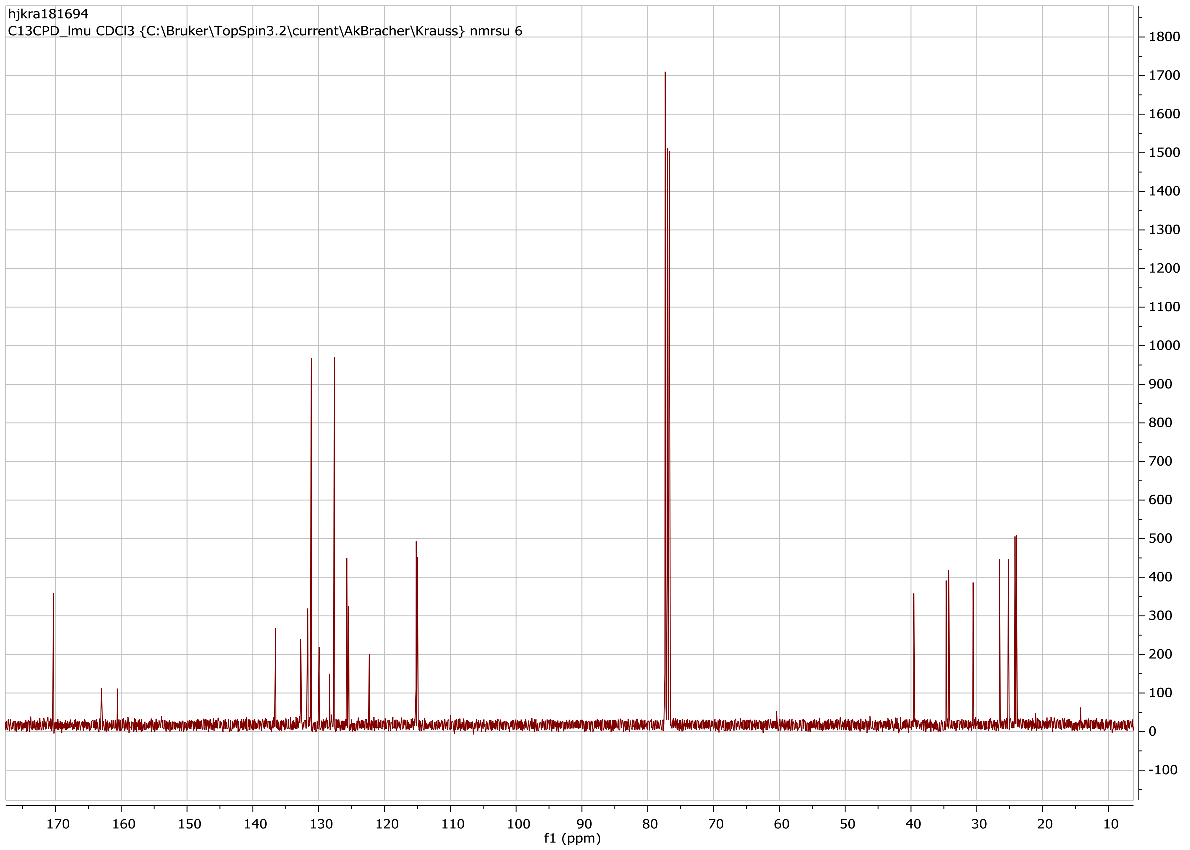

(*S*)-6-(2-(2-(4-Fluorophenyl)-5-isopropyl-3-phenyl-1*H*-pyrrol-1-yl)ethyl)-5,6-dihydro-2*H*-pyran-2-one (7)

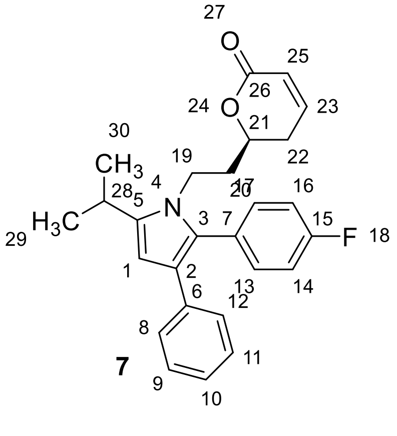

7

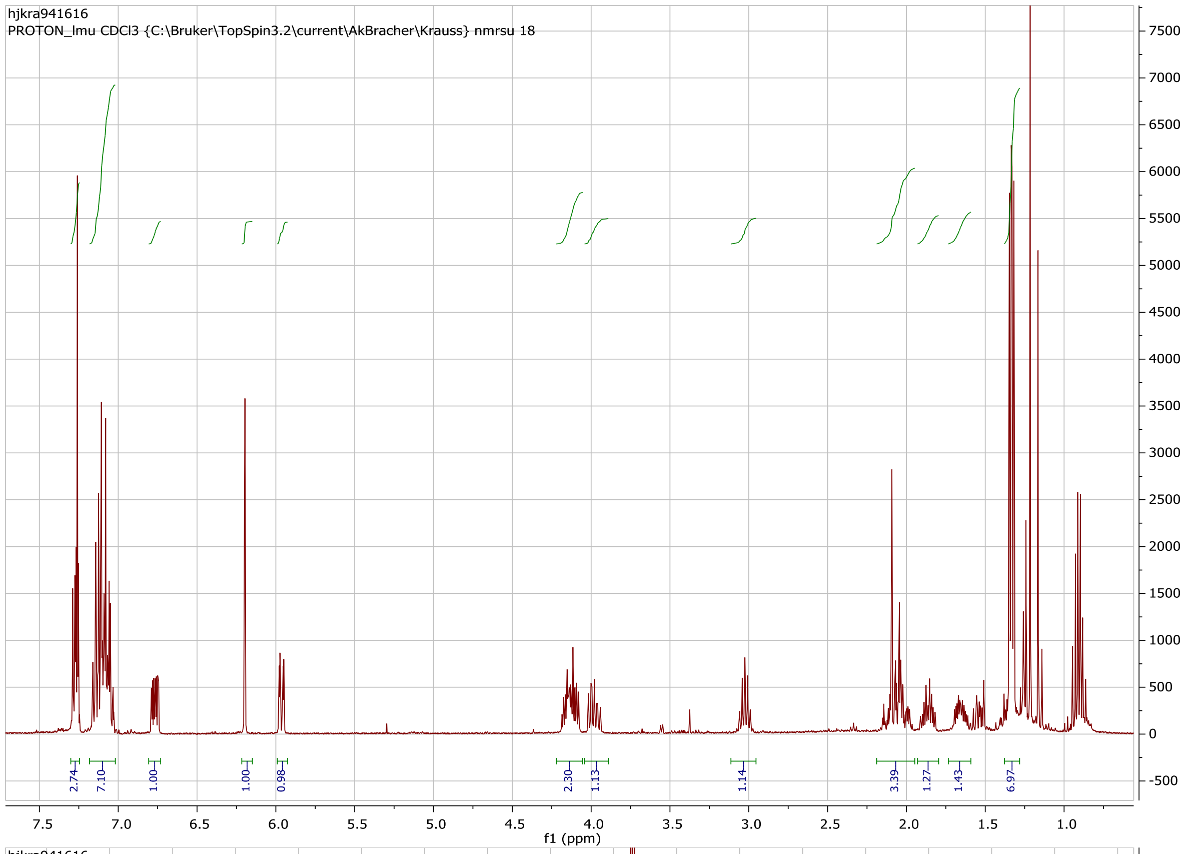
hjkra941616
PROTON_lmu CDCl3 {C:\Bruker\TopSpin3.2\current\AkBracher\Krauss} nmrsu 18
2.74
7.10
1.00
1.00
0.98
2.30
1.13
1.14
3.39
1.27
1.43
6.97
f1 (ppm)

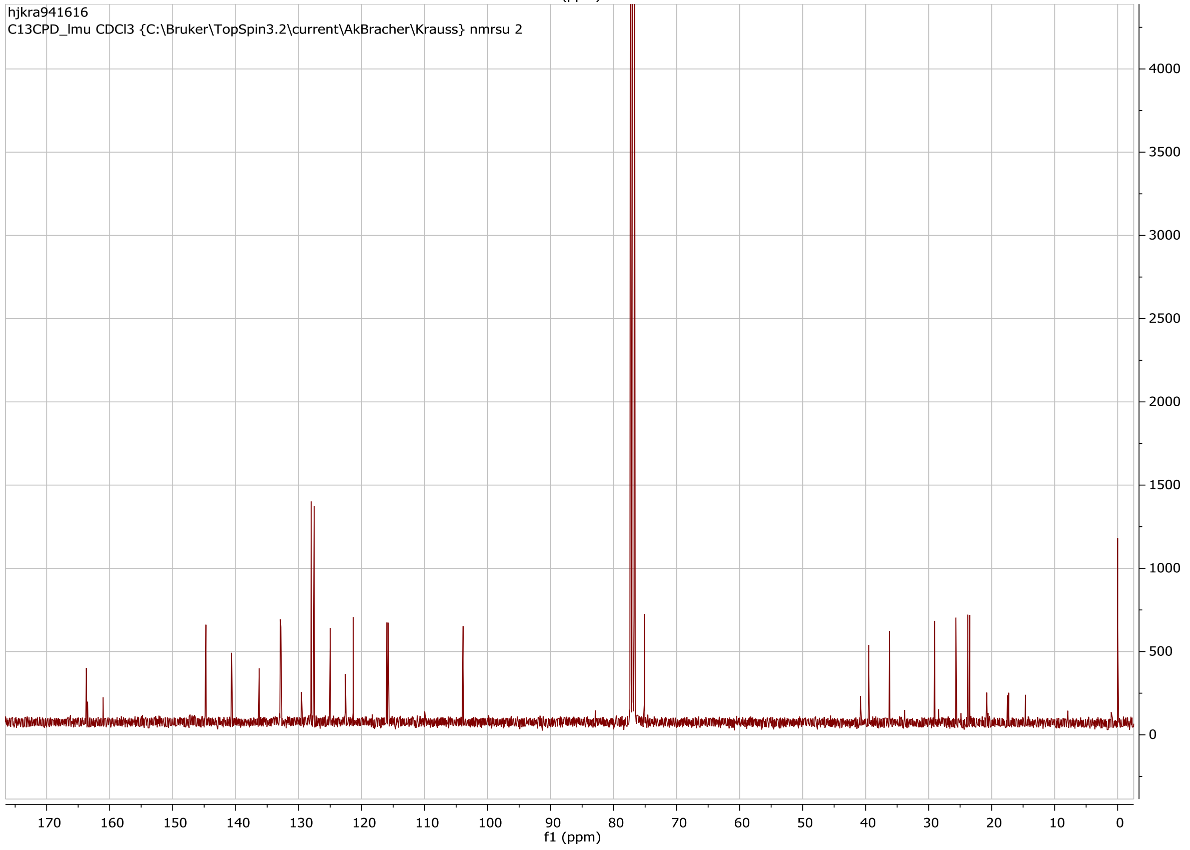
hjkra941616
C13CPD_lmu CDCl3 {C:\Bruker\TopSpin3.2\current\AkBracher\Krauss} nmrsu 2
f1 (ppm)

References

[1] J. Stach, J. Havlíček, L. Plaček, S. Rádl, Synthesis of some impurities and/or degradation products of atorvastatin, Coll. Czech. Chem. Commun. 73 (2008) 229-246. https://doi.org/10.1135/cccc20080229.

[2] Bruker (2012). *SAINT*. Bruker AXS Inc., Madison, Wisconsin, USA.

[3] G.M. Sheldrick (1996). *SADABS.* University of Göttingen, Germany.

[4] G.M. Sheldrick, SHELXT- Integrated space-group and crystal-structure determination, Acta Crystallogr. A, Found. Adv. 71 (2015) 3-8. http://search.ebscohost.com/login.aspx?direct=true&db=a9h&AN=100256836&site=ehost-live.

[5] L. Farrugia, WinGX and ORTEP for Windows: an update, J. Appl. Crystallogr. 45 (2012) 849-854. https://doi.org/10.1107/S0021889812029111.
