# Supplementary material for: Characterization of two new degradation products of atorvastatin calcium formed upon treatment with strong acids
Source: Beilstein J Org Chem. 2019 Sep 2;15:2085–91. doi: 10.3762/bjoc.15.206 (PMC6753681; doi:10.3762/bjoc.15.206)

BEILSTEIN JOURNAL OF ORGANIC CHEMISTRY

Supporting Information

for

Characterization of two new degradation products of atorvastatin calcium formed upon treatment with strong acids

Jürgen Krauß, Monika Klimt, Markus Luber, Peter Mayer and Franz Bracher

Beilstein J. Org. Chem. **2019,** *15,* 2085–2091. doi:10.3762/bjoc.15.206

checkCIF/PLATON report (Structure factors for artefacts 6 (wq033) and 7 (wv633))

License and Terms: This is a supporting information file under the terms of the Creative Commons Attribution License (http://creativecommons.org/licenses/by/4.0). Please note that the reuse, redistribution and reproduction in particular requires that the authors and source are credited.
The license is subject to the *Beilstein Journal of Organic Chemistry* terms and conditions: (https://www.beilstein-journals.org/bjoc)

checkCIF/PLATON report

Structure factors have been supplied for datablock(s) wq033, wv633

THIS REPORT IS FOR GUIDANCE ONLY. IF USED AS PART OF A REVIEW PROCEDURE FOR PUBLICATION, IT SHOULD NOT REPLACE THE EXPERTISE OF AN EXPERIENCED CRYSTALLOGRAPHIC REFEREE.

No syntax errors found. CIF dictionary Interpreting this report

Datablock: wq033

Bond precision: C-C = 0.0081 A Wavelength=0.71073

Cell:	a=9.6001(8)	b=17.4122(16)	c=38.547(4)
	alpha=90	beta=90	gamma=90
Temperature:	296 K		

	Calculated	Reported
Volume	6443.5(10)	6443.5(10)
Space group	P 21 21 21	P 21 21 21
Hall group	P 2ac 2ab	P 2ac 2ab
Moiety formula	C26 H26 F N O2	C26 H26 F N O2
Sum formula	C26 H26 F N O2	C26 H26 F N O2
Mr	403.48	403.48
Dx,g cm-3	1.248	1.248
Z	12	12
Mu (mm-1)	0.084	0.084
F000	2568.0	2568.0
F000’	2569.21	
h,k,lmax	11,20,46	11,20,46
Nref	11798[6566]	11793
Tmin,Tmax	0.997,0.998	0.960,1.000
Tmin’	0.992	

Correction method= # Reported T Limits: Tmin=0.960 Tmax=1.000
AbsCorr = MULTI-SCAN

Data completeness= 1.80/1.00 Theta(max)= 25.350

R(reflections)= 0.0667(7567) wR2(reflections)= 0.1283(11793)

S = 1.038 Npar= 818

The following ALERTS were generated. Each ALERT has the format
test-name_ALERT_alert-type_alert-level.
Click on the hyperlinks for more details of the test.

Alert level C

```
RINTA01_ALERT_3_C  The value of Rint is greater than 0.12
            Rint given   0.138
STRVA01_ALERT_4_C           Flack test results are ambiguous.
           From the CIF: _refine_ls_abs_structure_Flack    0.500
           From the CIF: _refine_ls_abs_structure_Flack_su    0.000
PLAT241_ALERT_2_C High   'MainMol' Ueq as Compared to Neighbors of        C43 Check
PLAT242_ALERT_2_C Low    'MainMol' Ueq as Compared to Neighbors of        C60 Check
PLAT242_ALERT_2_C Low    'MainMol' Ueq as Compared to Neighbors of        C64 Check
PLAT334_ALERT_2_C Small Aver. Benzene C-C Dist C47      -C52            1.37 Ang.
PLAT340_ALERT_3_C Low Bond Precision on  C-C Bonds ...............   0.00809 Ang.
PLAT906_ALERT_3_C Large K Value in the Analysis of Variance ......     4.668 Check
PLAT910_ALERT_3_C Missing # of FCF Reflection(s) Below Theta(Min).         6 Note
```

Alert level G

```
PLAT020_ALERT_3_G The Value of Rint is Greater Than 0.12 .........     0.138 Report
PLAT033_ALERT_4_G Flack x Value Deviates > 3.0 * sigma from Zero .     0.500 Note
PLAT791_ALERT_4_G Model has Chirality at C3         (Chiral SPGR)          R Verify
PLAT791_ALERT_4_G Model has Chirality at C5         (Chiral SPGR)          R Verify
PLAT791_ALERT_4_G Model has Chirality at C29        (Chiral SPGR)          R Verify
PLAT791_ALERT_4_G Model has Chirality at C31        (Chiral SPGR)          R Verify
PLAT791_ALERT_4_G Model has Chirality at C55        (Chiral SPGR)          R Verify
PLAT791_ALERT_4_G Model has Chirality at C57        (Chiral SPGR)          R Verify
PLAT883_ALERT_1_G No Info/Value for _atom_sites_solution_primary .     Please Do !
PLAT913_ALERT_3_G Missing # of Very Strong Reflections in FCF ....         1 Note
PLAT916_ALERT_2_G Hooft y and Flack x Parameter Values Differ by .      0.19 Check
PLAT978_ALERT_2_G Number C-C Bonds with Positive Residual Density.         1 Info
```

```
   0 ALERT level A = Most likely a serious problem - resolve or explain
   0 ALERT level B = A potentially serious problem, consider carefully
   9 ALERT level C = Check. Ensure it is not caused by an omission or oversight
  12 ALERT level G = General information/check it is not something unexpected

   1 ALERT type 1 CIF construction/syntax error, inconsistent or missing data
   6 ALERT type 2 Indicator that the structure model may be wrong or deficient
   6 ALERT type 3 Indicator that the structure quality may be low
   8 ALERT type 4 Improvement, methodology, query or suggestion
   0 ALERT type 5 Informative message, check
```

Datablock: wv633

Bond precision: C-C = 0.0022 A   Wavelength=0.71073

Cell:	a=9.7398(4)	b=10.4787(5)	c=20.4221(9)
	alpha=90	beta=90	gamma=90
Temperature:	112 K		

	Calculated	Reported
Volume	2084.29(16)	2084.29(16)
Space group	P 21 21 21	P 21 21 21
Hall group	P 2ac 2ab	P 2ac 2ab
Moiety formula	C26 H26 F N O2	C26 H26 F N O2
Sum formula	C26 H26 F N O2	C26 H26 F N O2
Mr	403.48	403.48
Dx,g cm-3	1.286	1.286
Z	4	4
Mu (mm-1)	0.087	0.087
F000	856.0	856.0
F000’	856.40	
h,k,lmax	16,17,34	16,17,34
Nref	10101[5594]	10078
Tmin,Tmax	0.993,0.995	0.960,0.990
Tmin’	0.991	

Correction method= # Reported T Limits: Tmin=0.960 Tmax=0.990
AbsCorr = MULTI-SCAN

Data completeness= 1.80/1.00   Theta(max)= 36.313

R(reflections)= 0.0524(8541)   wR2(reflections)= 0.1307(10078)

S = 1.082   Npar= 273

The following ALERTS were generated. Each ALERT has the format
test-name_ALERT_alert-type_alert-level.
Click on the hyperlinks for more details of the test.

Alert level C

```
PLAT220_ALERT_2_C Non-Solvent  Resd 1  C   Ueq(max)/Ueq(min) Range        4.3 Ratio
PLAT222_ALERT_3_C Non-Solv.  Resd 1  H   Uiso(max)/Uiso(min) Range        4.6 Ratio
PLAT910_ALERT_3_C Missing # of FCF Reflection(s) Below Theta(Min).          7 Note
PLAT911_ALERT_3_C Missing FCF Refl Between Thmin & STh/L=    0.600          8 Report
```

Alert level G

```
PLAT019_ALERT_1_G _diffrn_measured_fraction_theta_full/*_max < 1.0      0.996 Report
PLAT791_ALERT_4_G Model has Chirality at C13         (Chiral SPGR)          S Verify
PLAT883_ALERT_1_G No Info/Value for _atom_sites_solution_primary .     Please Do !
PLAT933_ALERT_2_G Number of OMIT Records in Embedded .res File ...         12 Note
PLAT978_ALERT_2_G Number C-C Bonds with Positive Residual Density.         22 Info
```

---

0 **ALERT level A** = Most likely a serious problem - resolve or explain
0 **ALERT level B** = A potentially serious problem, consider carefully
4 **ALERT level C** = Check. Ensure it is not caused by an omission or oversight
5 **ALERT level G** = General information/check it is not something unexpected

2 ALERT type 1 CIF construction/syntax error, inconsistent or missing data

```
3 ALERT type 2 Indicator that the structure model may be wrong or deficient
3 ALERT type 3 Indicator that the structure quality may be low
1 ALERT type 4 Improvement, methodology, query or suggestion
0 ALERT type 5 Informative message, check
```

It is advisable to attempt to resolve as many as possible of the alerts in all categories. Often the minor alerts point to easily fixed oversights, errors and omissions in your CIF or refinement strategy, so attention to these fine details can be worthwhile. In order to resolve some of the more serious problems it may be necessary to carry out additional measurements or structure refinements. However, the purpose of your study may justify the reported deviations and the more serious of these should normally be commented upon in the discussion or experimental section of a paper or in the "special_details" fields of the CIF. checkCIF was carefully designed to identify outliers and unusual parameters, but every test has its limitations and alerts that are not important in a particular case may appear. Conversely, the absence of alerts does not guarantee there are no aspects of the results needing attention. It is up to the individual to critically assess their own results and, if necessary, seek expert advice.

Publication of your CIF in IUCr journals

A basic structural check has been run on your CIF. These basic checks will be run on all CIFs submitted for publication in IUCr journals (*Acta Crystallographica*, *Journal of Applied Crystallography*, *Journal of Synchrotron Radiation*); however, if you intend to submit to *Acta Crystallographica Section C* or *E* or *IUCrData*, you should make sure that full publication checks are run on the final version of your CIF prior to submission.

Publication of your CIF in other journals

Please refer to the *Notes for Authors* of the relevant journal for any special instructions relating to CIF submission.

PLATON version of 17/03/2019; check.def file version of 04/03/2019

Datablock wq033 - ellipsoid plot

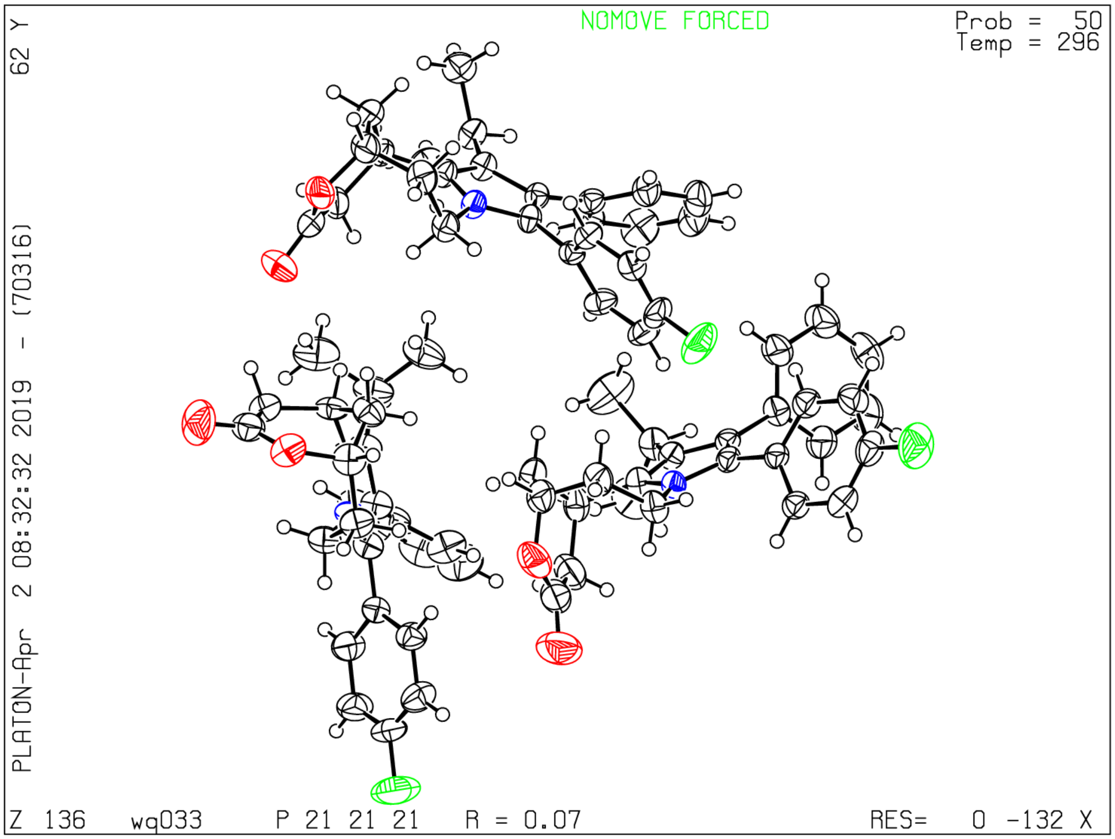

Datablock wv633 - ellipsoid plot

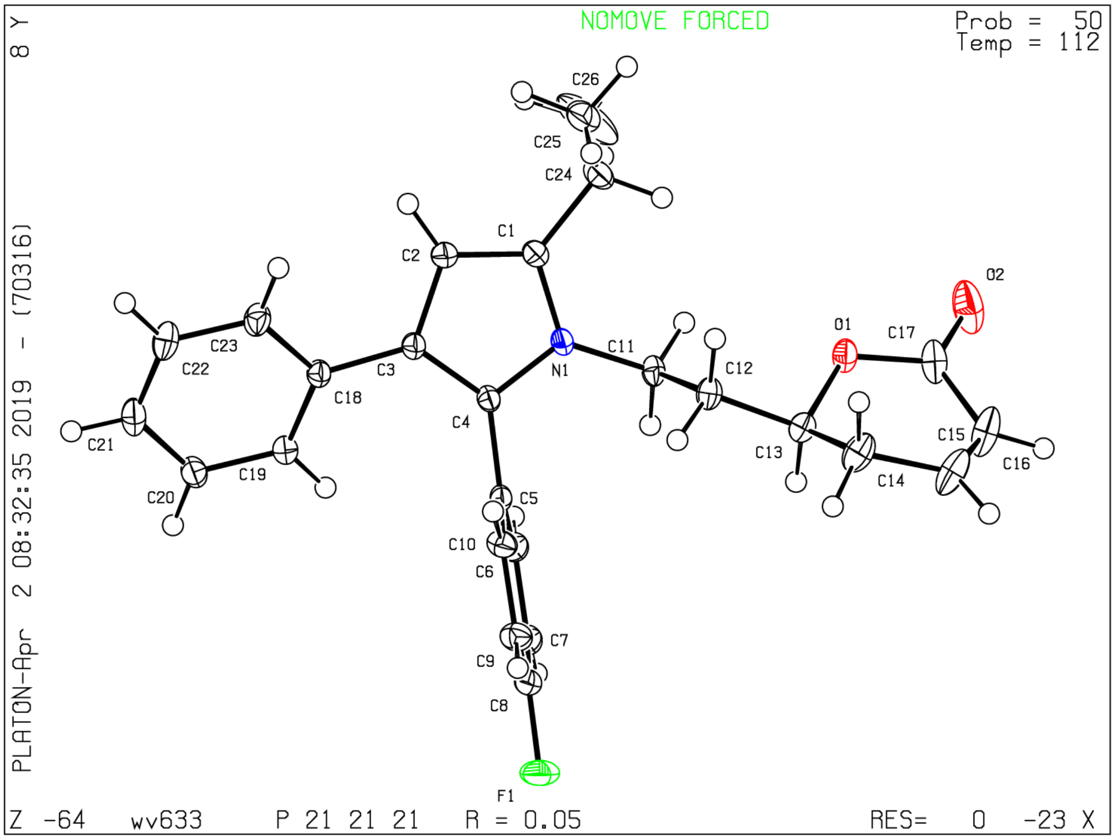

Supplement: File 2 — checkCIF/PLATON report (Structure factors for artefacts 6 (wq033) and 7 (wv633)). [file Beilstein_J_Org_Chem-15-2085-s002.pdf]
